# Corneal Amiodarone Deposition and Its Implications for Autologous Serum Eye Drop Therapy: A Case Report

**DOI:** 10.7759/cureus.96670

**Published:** 2025-11-12

**Authors:** Khushi Saigal, Shawn Sussmane, Sonal Tuli

**Affiliations:** 1 Ophthalmology, University of Florida, Gainesville, USA

**Keywords:** amiodarone keratopathy, autologous serum eye drops, corneal drug deposition, dry-eye disease, ocular surface toxicity

## Abstract

Corneal toxicity and deposits can develop from a variety of systemic and topical medications used in ophthalmic and non-ophthalmic care. We report a case of a 69-year-old man with dry eye syndrome on long-term amiodarone therapy who developed bilateral corneal verticillata shortly after initiating autologous serum eye drops (ASEDs). Despite improvement in epithelial healing, the new deposits raised concern for exacerbation of amiodarone-related corneal toxicity, possibly influenced by ASEDs. This case underscores the potential interaction between systemic therapy and ASEDs, highlighting the importance of careful monitoring in patients with ocular surface disease receiving medications known to cause ocular complications.

## Introduction

Dry eye syndrome (DES) is a complex disorder, causing ocular discomfort through symptoms like burning, itching, dryness, a foreign body sensation, and blurred vision. It is also marked by tear film instability, increased osmolarity, inflammation, and damage to the corneal and conjunctival epithelium. DES affects 5%-35% of the global population, with a higher incidence in females and variation by age, environmental conditions, systemic diseases, and medications [1].

The goal of DES treatment is to improve tear film stability, alleviate inflammation, and prevent complications [2]. Autologous serum eye drops (ASEDs) are a therapeutic approach using a patient's own diluted blood serum as a tear substitute. They contain growth factors, cytokines, vitamins, antioxidants, and other bioactive molecules that promote ocular surface repair. However, systemic medications present in the serum may also influence the ocular surface. Indeed, prior studies have demonstrated that systemically administered agents can be detected in ASEDs, including cyclosporine and mycophenolic acid in patients with severe ocular graft-versus-host disease [3]. Although detected at subtherapeutic concentrations, these findings raise the possibility that ASEDs can serve as a vehicle for systemic drug transfer to the ocular surface. In one report, corneal epitheliopathy worsened in a patient receiving ASEDs during HER2-targeted therapy, resolving only after ASEDs were withheld [4].

Immune checkpoint inhibitors, such as pembrolizumab and nivolumab, have revolutionized cancer treatment but are associated with rare ocular toxicities, from blurred vision to permanent vision loss. Similarly, amiodarone can cause corneal epitheliopathy leading to visual disturbances. It is still unclear whether systemic medications are introduced to the ocular surface through ASEDs and at what levels. These drugs provide examples of how systemic medications can manifest as ocular surface complications, a principle relevant to the hypothesis that ASEDs may deliver such agents directly to the cornea [5].

Corneal verticillata refers to whorl-like opacities in the corneal epithelium. This pattern is most commonly caused by cationic amphiphilic drugs such as amiodarone, which follow the natural centripetal migration of epithelial cells from the limbus toward the center of the cornea. Although often asymptomatic, verticillata may cause glare, halos, or blurred vision in some patients [6]. The lipophilicity of amiodarone and its metabolite promotes epithelial uptake and lysosomal accumulation, providing a plausible mechanism for drug deposition when reintroduced via ASEDs [7].

We present a case of a patient with severe DES on amiodarone who presented with severe corneal verticillata after using ASEDs, which may have exacerbated amiodarone ocular deposition. This case underscores the importance of clinician awareness that systemic medications can manifest as ocular findings, and that ASEDs may reintroduce such drugs directly to the ocular surface, amplifying their effects.

## Case presentation

A 69-year-old man was referred to the ophthalmology clinic from an outside eye care provider for progressively worsening vision in both eyes. His past medical history included long-term amiodarone use for recurrent arrhythmias. He had been taking amiodarone 400 mg orally once daily, with the dose increased 18 months prior due to poor arrhythmia control and failure of cardioversion. Cumulative dose and serum levels were not available.

On presentation, his best-corrected visual acuity (BCVA) was 20/40 in both eyes (OU). He reported a foreign body sensation and irritation in the left eye (OS). Examination revealed dermatochalasis, pingueculae, moderate punctate epithelial keratopathy through fluorescein staining, and mild nuclear sclerosis (OU). He worked in a sawmill but said he wore eye protection. At the time, he was on Tobradex QID for one week.

He was diagnosed with bilateral blepharitis and dry eye disease. Conservative management prescribed included warm compresses, lid scrubs, preservative-free artificial tears, and olopatadine for a possible allergic component. The Tobradex was discontinued due to suspected toxicity. He also showed signs of floppy eyelid syndrome, prompting a recommendation for sleep apnea testing.

At the one-month follow-up visit, BCVA remained 20/40 OD but had worsened to 20/80 OS. He was advised to use preservative-free artificial tears six to eight times daily and a lubricating ointment at night. He admitted to using brimonidine 0.025% for eye redness, which was discontinued, as were the preserved tears. His cataracts were deemed clinically insignificant and not considered responsible for his decreased vision. The timeline of discontinuation of these potential confounders preceded the appearance of whorl-like corneal deposits by approximately three months.

At a subsequent visit, BCVA improved to 20/20 OD and 20/40 OS, though the patient reported increased discomfort, particularly in the mornings and evenings, with difficulty reading fine print. He was advised to continue artificial tears, eyelid scrubs, and baby shampoo for lid hygiene and started on ASEDs 20%, one drop in each eye four times daily. The ASEDs were prepared according to standard protocol: blood allowed to clot, centrifuged, diluted with sterile saline, frozen in individual bottles, and thawed overnight before use, with only one bottle thawed at a time. Adherence was confirmed at follow-up visits. Oral doxycycline was also started.

**Figure 1:**
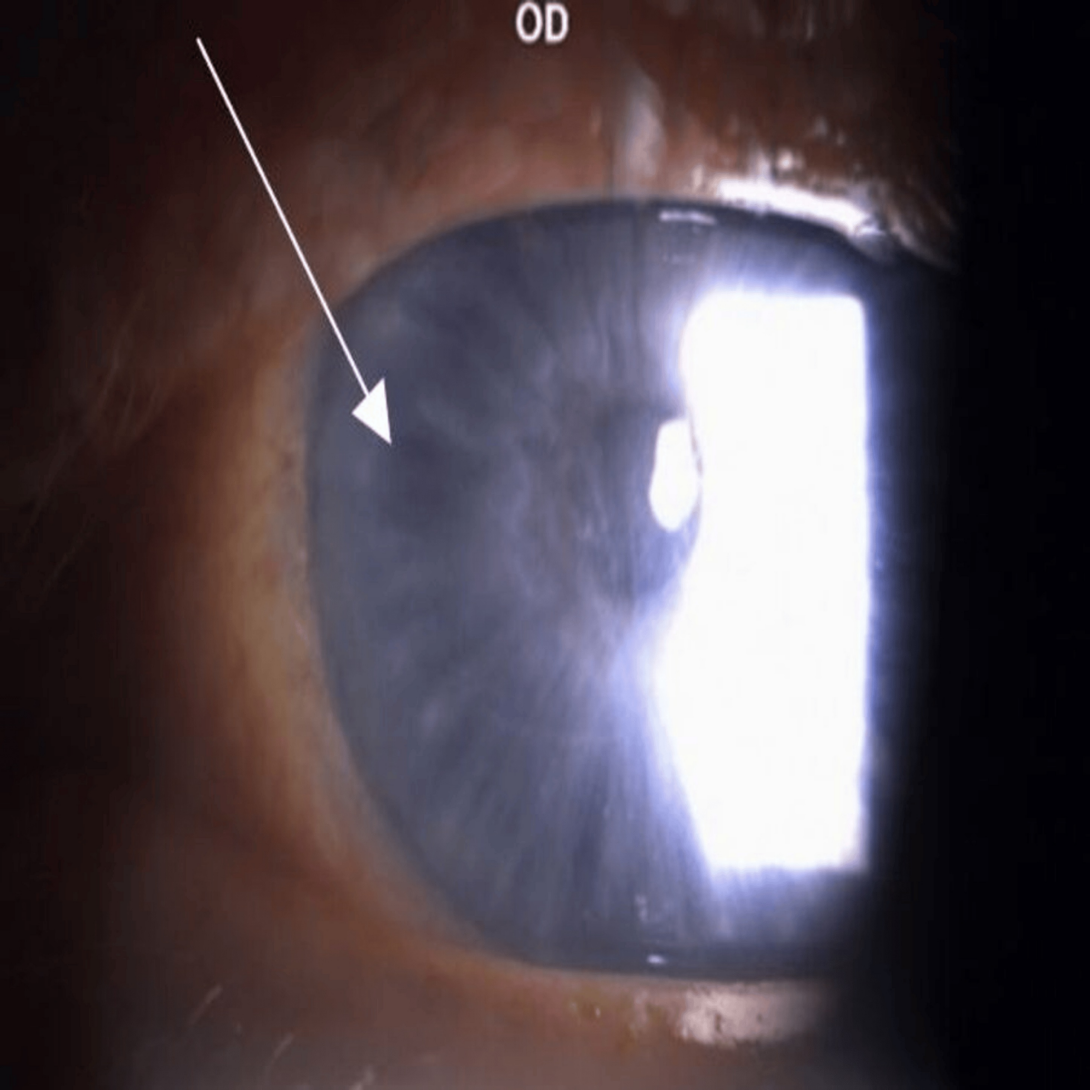
Right eye corneal verticillata Slit-lamp photograph of the right eye (OD) demonstrating prominent whorl-like corneal epithelial deposits consistent with corneal verticillate (indicated with arrows). The slit-lamp image was obtained using a Topcon SL-D701 biomicroscope at approximately 16× magnification, consistent with standard anterior segment imaging.

**Figure 2:**
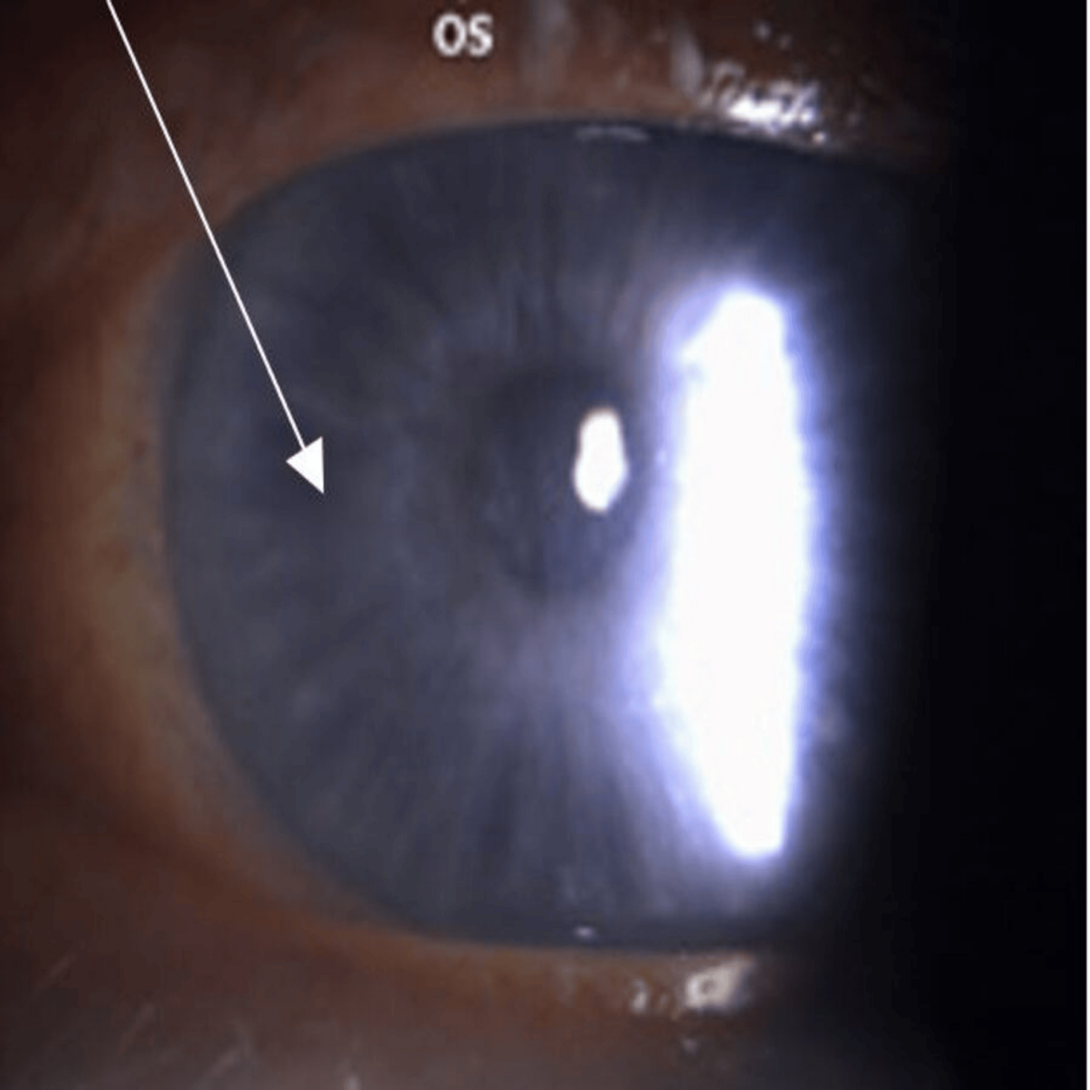
Left eye corneal verticillata Slit-lamp photograph of the left eye (OS) showing similar whorl-like corneal epithelial deposits (indicated with arrows). At four-month follow-up, best-corrected visual acuity (BCVA) was 20/20 OD and 20/60 OS. The corneal epithelium in the OD had healed completely, while punctate keratopathy in the OS had significantly improved. Both eyes, however, developed these characteristic whorl-like deposits. The slit-lamp image was obtained using a Topcon SL-D701 biomicroscope (Topcon Corporation, Tokyo, Japan) at approximately 16× magnification, consistent with standard anterior segment imaging.

At the four-month follow-up, BCVA was 20/20 OD and 20/60 OS. Slit-lamp examination revealed that the corneal epithelium OD had healed completely, and the OS punctate keratopathy had significantly improved. Both eyes had, however, developed significant whorl-like epithelial deposits (Figures 1, 2).

Corneal verticillata refers to whorl-like opacities in the corneal epithelium caused by cationic amphiphilic drugs such as amiodarone, which accumulate in epithelial cells as they migrate centripetally from the limbus to the corneal center. While often asymptomatic, verticillata may produce glare, halos, or blurred vision. Amiodarone cessation was not an option due to his need for arrhythmia control, so ASEDs were continued with monitoring for deposit regression. The patient was reassured that verticillata is often not visually significant, and the poor vision OS was likely due to incomplete epithelial healing in the left eye, with improvement expected similar to the right eye, which had healed completely. Debridement or phototherapeutic keratectomy was discussed but not pursued, as it was thought that post-procedure serum drops could cause further deposition.

**Figure 3:**
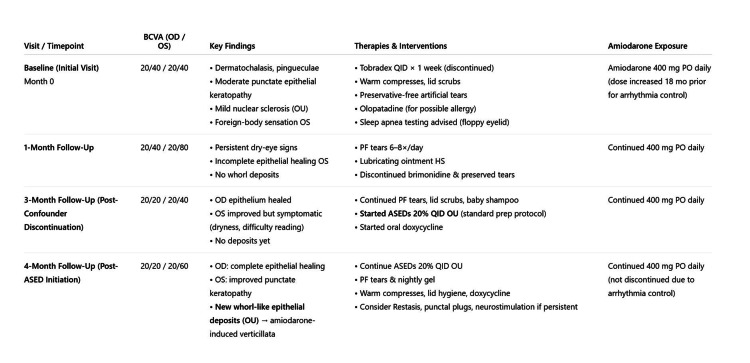
Timeline of clinical course and development of corneal verticillata Schematic timeline summarizing the patient’s clinical course, including initial presentation, initiation of autologous serum eye drop (ASED) therapy, and subsequent development of corneal verticillata. Best-corrected visual acuity (BCVA) changes and slit-lamp findings are illustrated over time.

Dry eye symptoms persisted despite treatment with artificial tears, nightly gel, warm compresses, eyelid wipes, and oral doxycycline. Other therapies like Restasis, punctal plugs, and neurostimulation were considered if the patient’s symptoms persisted. Potential confounders, including occupational exposure to sawdust, prior brimonidine use, and preserved tear use, were considered but unlikely to account for the observed whorl-like epithelial deposits given the timeline of discontinuation and the characteristic appearance of verticillata. The patient’s clinical timeline is shown in Figure 3.

## Discussion

This case describes a patient on amiodarone therapy who developed corneal verticillata, potentially significantly worsened by concurrent use of ASEDs. To our knowledge, this is the first report of this finding.

Amiodarone is an effective antiarrhythmic medication for atrial fibrillation [8], but its use is limited by adverse effects, including ophthalmologic complications. Ocular side effects typically appear after an average of 15.3 months of therapy [8]. Amiodarone’s metabolite, N-desethylamiodarone, is associated with corneal verticillata [6,9]. The deposits present as a distinct, linear, arborizing pattern within the epithelium, typically not extending beyond the basal membrane, and are generally bilateral and symmetrical [10].

ASEDs are commonly used in DES management by mimicking natural tears, promoting ocular surface healing, and potentially mitigating ocular surface toxicity from topical medications. A study showed that 20% autologous serum improved corneal epithelial integrity and stability in patients with toxic corneal epitheliopathy from benzalkonium chloride-containing anti-glaucoma medications [11]. This suggests ASEDs may counteract ocular surface damage from topical medications.

Further studies suggest that systemic medications may accumulate in ASEDs, with detectable levels of cyclosporine and mycophenolic acid found in ASEDs in patients with severe ocular graft-versus-host disease [3]. Although these concentrations were below therapeutic ranges, the effect on ASED therapy remains unclear. In one case, a patient with HER2-positive breast cancer developed worsening corneal epitheliopathy after starting A166 therapy, which persisted until ASEDs were withheld due to potential contamination [4].

Given that cyclosporine, mycophenolic acid, and A166 have been detected in ASEDs, we hypothesize that amiodarone could also be present at clinically relevant levels, potentially contributing to the severe corneal verticillata in our patient. The diffuse presentation may have arisen from a combination of systemic medication being secreted in the tear film and released from limbal vessels, and direct exposure of the ocular surface to topical amiodarone in the ASEDs, leading to rapid and intense corneal deposition in the healing epithelial cells.

Although the patient was on a stable daily dose of amiodarone (400 mg orally), no direct measurements of amiodarone were obtained in serum, tears, or the ASEDs, and no dechallenge or dose-response assessment was performed. Other potential keratotoxic exposures, including prior Tobradex, brimonidine, and preserved tears, were discontinued prior to the appearance of verticillata, and occupational sawmill exposure was minimal with eye protection. These factors make causality uncertain, and this observation should be considered hypothesis-generating.

Managing this patient is complex as he is dependent on both amiodarone and ASEDs for his atrial fibrillation and epithelial health, respectively. The treatment plan includes continuing ASED therapy with monitoring for improvement in vision anticipated with improvement in the ocular surface.

This case highlights the potential for ASEDs to influence ocular drug deposition when used with systemic therapy, raising concerns about their role in exacerbating corneal complications. While causality cannot be established, structured follow-up with objective corneal assessments (e.g., slit-lamp grading, densitometry) and future quantification of systemic drug levels in tears or ASEDs would be valuable to clarify potential mechanisms of drug deposition and guide clinical recommendations.

We recommend that future studies explore whether amiodarone levels in ASEDs correlate with verticillata severity, and whether brief, monitored modifications of ASED therapy could inform management strategies.

## Conclusions

This case suggests a potential interaction between autologous serum eye drops (ASEDs) and systemic medications that could influence ocular drug deposition and contribute to corneal complications. In our patient, the temporal association between long-term amiodarone use and the development of corneal verticillata after initiating ASEDs raises the possibility that drug accumulation within the serum drops may have played a contributory role. Although amiodarone’s ocular toxicity is well recognized, the possible modulation of this effect by ASEDs warrants further exploration. This case illustrates the complexity of managing patients who require both systemic and ocular surface therapies and underscores the importance of multidisciplinary coordination between cardiologists and ophthalmologists. Given the established benefits of ASEDs in severe ocular surface disease and the absence of confirmatory drug measurements or a dechallenge in this case, clinicians should exercise heightened vigilance, ensure informed consent regarding the theoretical risk of enhanced drug deposition, and consider careful patient selection, close monitoring, and concentration adjustments or alternative tear substitutes in individuals receiving systemic agents known to deposit in the cornea. Building upon this observation, we are planning future studies to examine whether amiodarone levels in ASEDs correlate with verticillata severity and to evaluate whether brief, monitored modifications of ASED therapy could inform management strategies.
